# Proximal Femur Locking Plate for Sub-Trochanteric Femur Fractures: Factors Associated with Failure

**DOI:** 10.2174/1874325001711011058

**Published:** 2017-08-29

**Authors:** Akbar Zubairi, Rizwan Haroon Rashid, Marij Zahid, Pervaiz Mahmood Hashmi, Shahryar Noordin

**Affiliations:** 1 Section of Orthopedics, Aga Khan University Hospital, Karachi, Pakistan; 2 Section of Orthopedics, Aga Khan University Hospital, Karachi, Pakistan; 3 Section of Orthopedics, Aga Khan University Hospital, Karachi, Pakistan

**Keywords:** Locking compress plate (LCP), Proximal locking screws, Implants, Osteoporosis, Plate-screw density, Stresses

## Abstract

**Introduction:**

Proximal femur locking compression plates (PF-LCP) have gained popularity since their inception due to superior biomechanical stability and durability but clinical experience has shown conflicting results including implant failure.

**Objective:**

To study the incidence of implant failure in patients with sub-trochanteric fractures managed with proximal femoral locking compression plate and identify potential risk factors associated with the failure.

**Materials & Methods:**

Fifty patients with sub-trochanteric fractures, operated upon with titanium PF-LCP were included in the study from January 2012 to December 2014. These plates were of two designs including one five 5.0 mm proximal locking screws (implant A) and other with three 6.5 mm proximal locking screws (implant B). Fractures were classified according to AO/OTA and Seinsheimer classification. Patients had regular follow-up visits for at least a year, allowing for clinical and radiological assessment of union and implant-related complications.

**Results:**

A total of 13 out of 50 (26%) plates failed of which 7 were implant fractures, 3 screw breakage and 3 screw cut outs. 70% of the failures occurred in elderly females. Overall implant failure was significantly more common in patients >50 years (p 0.04). Comparing the two different designs of implants used, implant A was more likely to fail at a plate screw density of 0.8 or more (p 0.02), whereas implant B was associated with significant failure when less than 4 proximal screws were used (p 0.03).

**Conclusion:**

This study revealed a high failure rate (26%) of this implant. Attention to the neck shaft angle difference, number of proximal screws and plate screw density may help reduce failure rates, particularly in elderly osteoporotic females.

## 1 INTRODUCTION

The fixation of sub-trochanteric fractures is challenging associated with high incidence of per-operative and post-operative complications and failure [1] as these fractures are subjected to higher biomechanical stresses with both compression and competing forces exerted by muscular attachments acting medially that tend to pull and mal-align the fractured fragment [2]. The etiology of sub-trochanteric fractures vary widely with high energy trauma being the most common in young adults while ground level fall in the elderly [3]. There has always been a controversy over the best implant option for these fractures. Conventional implants include dynamic hip screw, dynamic condylar screw, angular blade plates and cephalo-medullary nails. Proximal femur locking compression plates (PF-LCP) have gained popularity over the last decade as a feasible option for fixation of fractures of the proximal femur. This is because of its pre-contoured shape, providing three dimensional fixation mechanical advantage and multi-angular stability with locking screws in the femoral head and simultaneously preserving bone stock especially in osteoporotic bone [4-9]. This PF-LCP has an advantage of a minimal incision and sliding of plate within sub-muscular plane avoiding potential morbidity of incision site like trochanteric pain and surgical site infections [1, 10-12].

Biomechanical studies have shown superior bio-mechanical stability and durability of the PF-LCP as compared to other contemporary implants available for fixation of proximal femur fractures [10, 13]. This provides less torsional stiffness and cyclic loading deformation [14]. Literature has shown importance of various biomechanical factors of the implant for successful outcome of LCP one of which is the plate screw density (number of screws inserted divided by the number of plate holes). Some studies have shown that this should be under 0.4 to 0.5 [15]. It is important that the number of screws inserted is small enough so as to keep the screw loading low. In the case of simple fractures where there is bone contact one or two combination holes should be left empty at the level of fracture so as to reduce the strain at the fracture site by allowing a larger area of stress distribution on the plate while in complex (comminuted) fractures with a lack of bone contact the holes closest to the fracture is recommended in the studies. Filling all screws holes lead to stress concentration and high strain which leads to cyclic loading and implant failure [4, 15].

Clinical experience with this implant has shown variable results with some studies reporting good functional and radiological outcomes whereas others have reported unacceptably high rates of complications and implant failure [1, 5, 16, 17]. Our goal was to define the factors that may be associated with failure of this implant even when performed by experienced surgeons in high volume centers [18, 19] as the literature is still unclear regarding such associations.

## 2 MATERIALS AND METHODS

It is a retrospective case-control study and included all the patients who underwent primary fixation of sub-trochanteric fractures with PF-LCP from 1^st^ Jan 2012 to 31^st^ Dec 2014 at our institute. Approval was granted from our institute’s Ethics review committee (3185-SUR-ERC-14). Peri-prosthetic, pathological fractures (other than osteoporosis), revision surgeries and patients with incomplete medical records and follow-up <1 year were excluded from the analysis.

**Fig. (1):**
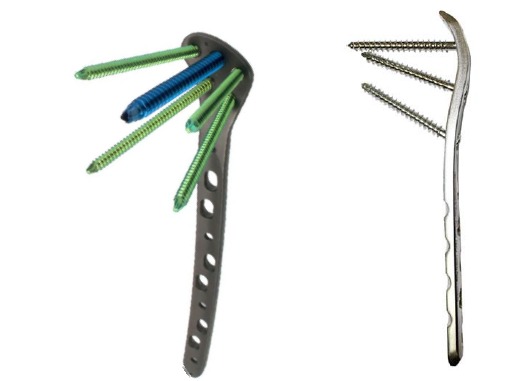
a) Implant A (Kanghui); b) Implant B (Double Medical).

All the patients were operated with patient in supine position on a traction table using a lateral approach. Two types of implant were used Implant A (Kanghui) with 5 proximal screws and Implant B (Double Medical) with 3 proximal screws. (Figs. **1a**, **b**) Data was collected on a structured performa. Patient’s demographic characteristics were recorded from patient records, along with findings at presentation and follow up. Radiographs were reviewed to classify fractures according to the AO fracture classification [20] and Seinsheimer classification [21] and to record radiological features of the fixation construct and identify implant failure. Patients were divided into two groups; cases (with implant failure) and control group (with union). Data was analyzed *via* IBM SPSS v22. Both the groups were analyzed for comparison. Logistic regression was used to evaluate the risk factors using Odds ratio at 95% confidence interval. Continuous variables were expressed as mean and categorical variables expressed as frequencies and percentages.

## 3 RESULTS

A total of 82 patients were operated with PF plate of which 32 were excluded according to the exclusion criteria. 50 patients were included in the study. Mean age of the patients was 58.18 years + 21.7 SD. Twenty-eight patients (56%) were females while 22 (44%) were male. The major cause of injury was low energy ground fall (64%) followed by road traffic accidents (34%) and fire arm injury (2%). Data was then cross tabulated into cases (with Implant failure) and controls (union or no implant failure). Both groups were comparable in terms of baseline characteristics (Table **1**). Thirteen patients (26%) experienced implant failures including 7 plate breakages and 3 cases each of screw breakage and cut-out. Implant characteristics like plate span ratio, number of proximal screws, plate screw density, femoral neck-shaft angle difference and implant types were also compared and analyzed. Mean femoral neck shaft angle (caput-column-diaphyseal angle) difference was -3.03 for the union group and -5.62 for the failure group showing statistically significant correlation between the two groups. The two implant designs A and B were compared and there were similar rates (26%) of failure between the two implants (Table **2**).

When the patients were stratified into two groups *i.e.* less than and more than 50 years of age there was a significant risk of failure in patients older than 50 years. When the number of proximal screws was analyzed separately for each implant design, implant A showed a significant vulnerability to failure if less than 4 screws were used proximally. Implant B was associated with increased risk of failure with increased plate screw density of 0.8 or more (Table **3**). All the patients were mobilized non-weight bearing at 6 weeks, partial weight bearing for further 6 weeks and full weight bearing after 12 weeks post-operatively. The mean time to union for those patients who achieve union was 6.4 months while a similar time to failure was also noted at 6.6 months. The characteristics of patients with implant failure and revision surgeries are shown in Table **4**.

## 4 DISCUSSION

Many studies regarding PF-LCP use in per-trochanteric fractures have been reported in the literature but only limited data has registered the outcomes in isolated sub-trochanteric fractures as these implants does not allow guided collapse in inter-trochanteric fractures and are not a promising option in the later. Zha *et al*. [5] reported study including 110 cases with per-trochanteric fractures and reported one case each of non-union and implant breakage and 2 cases of infection. Johnson *et al*. [22] reported 41.4% failure rates of which 83% patients were elderly females. Gunadham *et al*. [23] reported 23% failure rate in a comparative analysis of 26 patients with sub-trochanteric fractures including 2 broken plates, 1 broken screw, 1 non-union and 2 varus collapse. Glassner *et al*. [18] reported a case series including 10 patients with implant failure in 7 cases (70%) with 2 plate and 2 screw breakages and loss of fixation from varus collapse and implant cut-out in 3 cases. The mean age of the patients was 56.4 years. Saini *et al*. [1] however reported no implant failure in their study including 32 patients with sub-trochanteric fractures although they reported 2 cases of delayed union and infection each, two patients with limb shortening and one with external rotation deformity. The current study showed 26% implant failure rate, with 7 cases of implant breakage and 3 cases each of screw breakage and screw cut-out with mean age of 58.18 years.

The significant association that have arisen from the current study include age greater than 50 years, and probably this might be linked to osteoporosis in this group but even so the locking implants are supposed to be designed for osteoporotic bone and are the best available from our arsenal for this patient group. Moreover only 4 patients underwent peri-operative DEXA scan and all of them revealed severe osteoporosis (T < -3.5). These are not considered one of the characteristic insufficiency fractures like the neck of femur fracture where routine bone mineral density is checked and osteoporosis treatment administered. Increased incidence of failure with less number of proximal screws in implant A could be due to the proximal hold that they provide in the osteopenic cancellous bone due to their smaller diameter. As these implants are anatomically pre-contoured with fixed directions of the locking screws, anatomic variability of the patients’ femur (neck size and anteversion) may result in limited number of proximal screw options. Similarly increased plate-screw density in implant B possibly leads to increased rigidity of construct, with increased stress and strain on the implant with cyclic loading resulting in failure of this implant.

**Fig. (2):**
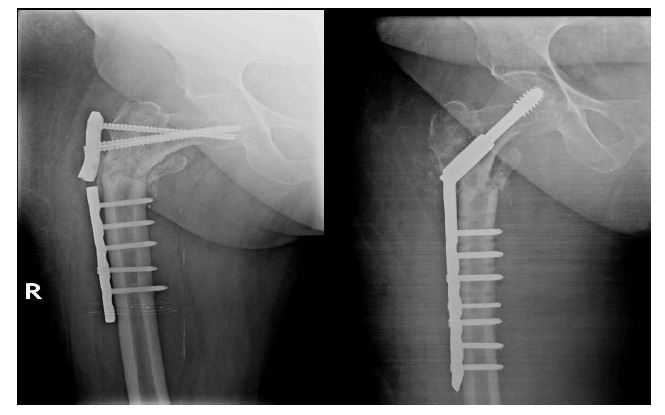
a) Post-operative radiograph of a 73 year old lady showing plate breakage due to varus collapse at 4months follow-up and fracture line crossing an empty hole ;b) Revision surgery with DHS.

**Fig. (3):**
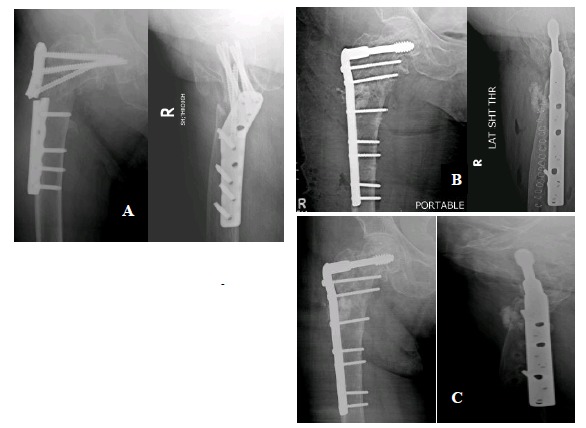
A) Varus collapse with PF-plate 18 months post-op, B) Immediate post-op radiographs after revision with DCS, C) Radiographs taken 6 months post revision showing good healing.

Femoral-neck shaft angle difference was yet another associated factor of significance. Inability to reconstruct neck shaft angle to within 5 degrees of opposite side led to increased risk of implant failure irrespective of the implant design. Glassner *et al*. [18] reported 3 out 7 implant failures due to varus collapse and cut-out. Wieser *et al*. [19] reported 4 cases of implant failure of which 2 cases showed varus mal-alignment on post-operative radiographs. (Figs. **2a**, **b**) This might be a reason for their failure. However Hossain MM *et al*. [24] reported no statistical difference between implant failure and femoral neck shaft angle. (Figs. **3a**, **b**).

The strength of the current study is a larger sample size as compared to other comparator studies with only isolated sub-trochanteric fractures included. Limitation of our study was its retrospective nature which did not allow us to delineate any association of osteoporosis with implant failure, an important avenue for future research. Considering high failure rates, early intervention with bone grafting may also be an option rather than wait for failure in high risk patients.

## CONCLUSION

The study revealed a high failure rate (26%) with use of PF-LCP in sub-trochanteric fractures, most common cause identified was the plate breakage. Attention to the number of proximal screws, plate-screw density and adjustment of neck shaft angle may help reduce the failure rates and particular care should be taken when using these implants, especially when going for fixation in elderly females.

## Figures and Tables

**Table 1:** Comparison between the two groups.

**Variable**	**Implant Failure**		***Significance***	
	**No**	**Yes**		
Age (mean)	56 + 23.0 SD	64.28 + 12.0 SD		0.104
Gender				
Male	18 (48.6%)	4 (30.8%)		0.273
Female	19 (51.4%)	9 (69.2%)		
Mechanism of injury				
Fall	23 (62.2%)	9 (69.23%)		
RTA	13 (35.1%)	4 (30.8%)		0.921
FAI	1 (2.7%)	0 (0%)		
Fracture classification				
AO				
A	6 (16.2%)	3 (23.1%)		
B	22 (59.5%)	4 (30.8%)		0.192
C	9 (24.3%)	6 (46.1%)		
Seinsheimer’s				
<3	24 (64.9%)	6 (46.2%)		0.236
>4	13 (35.1%)	7 (53.8%)		
ASA status				
I	5 (13.5%)	2 (15.4%)		
II	21 (56.8%)	8 (61.5%)		0.796
III	11 (29.7%)	3 (23.1%)		

RTA= Road traffic accident; FAI= Fire arm injury

**Table 2:** Radiological evaluation of implant.

**Variable**	**Implant Failure**		***Significance***
	**No**	**Yes**	
Proximal screws (mean)	4.16 + 0.65 SD	3.46+1.13SD	0.21
Plate screw density (mean)	0.70 + 0.11 SD	0.74 + 0.15 SD	0.29
Plate-span ratio (mean)	3.21 + 1.34 SD	3.14+1.33 SD	0.87
Neck-shaft angle difference (mean)	-3.03 + 0.38 SD	-5.62 + 4.073 SD	0.007
Posterio-medial buttress reconstruction	2 (5.4%)	1 (7.7%)	0.77
Type of plate			0.99
Implant A (Kanghui)	20 (74%)	7 (26%)	
Implant B (Double Medical)	17 (73.9%)	6 (26.1%)	

**Table 3:** Significant associations.

**Variable**	**Implant Failure**		***Significance***
	**No**	**Yes**	
Age			
<50 years	17 (46%)	2 (15%)	0.049
>50 years	20 (54%)	11 (85%)	
Proximal Screws (mean ± SD)			
Implant A	4.40 ± 0.60	3.57 ± 1.40	0.04
Implant B	3.88 ± 0.60	3.33 ± 0.82	
Plate-screw density (mean ± SD)			
Implant A	0.70 ± 0.12	0.70 ± 0.20	0.99
Implant B	0.79± 0.03	0.71± 0.08	0.026
Neck-shaft angle difference	-3.03 ± 0.38 SD	-5.62 ± 4.07 SD	0.007
(mean ± SD)			

**Table 4:** Implant failure characteristics.

**Age/ Sex**		**Type of Plate**	**Type of Failure**	**Time to Failure (in months)**	**Revision**
60 M		A	Screw cut out	2	THR
60 F		A	Plate breakage	2	THR
54 F		B	Screw breakage	6	Re-fixation with PF-LCP
82 F		B	Plate breakage	14	Data not available
75 F		A	Screw breakage	4	DHS
60 M		B	Screw cut out	2	Data not available
54 F		A	Screw cut out	2	Data not available
48 M		A	Plate breakage	12	DHS
46 M		A	Plate breakage	10	Data not available
73 F		B	Plate breakage	8	DHS
80 F		A	Screw breakage	4	Re-fixation with PF-LCP
65 F		B	Plate breakage	9	IM Nail
78 F		B	Plate breakage	9	DCS

THR= Total Hip Replacement; DHS= Dynamic Hip Screw; IM= Intra-medullary; DCS= Dynamic Compression Plate
